# Epigenetic and Transcriptomic Regulation Landscape in HPV+ Cancers: Biological and Clinical Implications

**DOI:** 10.3389/fgene.2022.886613

**Published:** 2022-06-14

**Authors:** Rosario Castro-Oropeza, Patricia Piña-Sánchez

**Affiliations:** Molecular Oncology Laboratory, Oncology Research Unit, Oncology Hospital, IMSS National Medical Center, Mexico City, Mexico

**Keywords:** HPV, epigenetic, therapy, viral oncoprotein, regulation, biomarker, miRNA, LncRNA

## Abstract

Human Papillomavirus (HPV) is an oncogenic virus that causes the highest number of viral-associated cancer cases and deaths worldwide, with more than 690,000 new cases per year and 342,000 deaths only for cervical cancer (CC). Although the incidence and mortality rates for CC are declining in countries where screening and vaccination programs have been implemented, other types of cancer in which HPV is involved, such as oropharyngeal cancer, are increasing, particularly in men. Mutational and transcriptional profiles of various HPV-associated neoplasms have been described, and accumulated evidence has shown the oncogenic capacity of E6, E7, and E5 genes of high-risk HPV. Interestingly, transcriptomic analysis has revealed that although a vast majority of the human genome is transcribed into RNAs, only 2% of transcripts are translated into proteins. The remaining transcripts lacking protein-coding potential are called non-coding RNAs. In addition to the transfer and ribosomal RNAs, there are regulatory non-coding RNAs classified according to size and structure in long non-coding RNAs (lncRNAs), circular RNAs (circRNAs), and small RNAs; such as microRNAs (miRNAs), piwi-associated RNAs (piRNAs), small nucleolar RNAs (snoRNAs) and endogenous short-interfering RNAs. Recent evidence has shown that lncRNAs, miRNAs, and circRNAs are aberrantly expressed under pathological conditions such as cancer. In addition, those transcripts are dysregulated in HPV-related neoplasms, and their expression correlates with tumor progression, metastasis, poor prognosis, and recurrence. Nuclear lncRNAs are epigenetic regulators involved in controlling gene expression at the transcriptional level through chromatin modification and remodeling. Moreover, disruption of the expression profiles of those lncRNAs affects multiple biological processes such as cell proliferation, apoptosis, and migration. This review highlights the epigenetic alterations induced by HPV, from infection to neoplastic transformation. We condense the epigenetic role of non-coding RNA alterations and their potential as biomarkers in transformation’s early stages and clinical applications. We also summarize the molecular mechanisms of action of nuclear lncRNAs to understand better their role in the epigenetic control of gene expression and how they can drive the malignant phenotype of HPV-related neoplasia. Finally, we review several chemical and epigenetic therapy options to prevent and treat HPV-associated neoplasms.

## 1 Epidemiology of Human Papillomavirus Neoplasms

**FIGURE 1:**
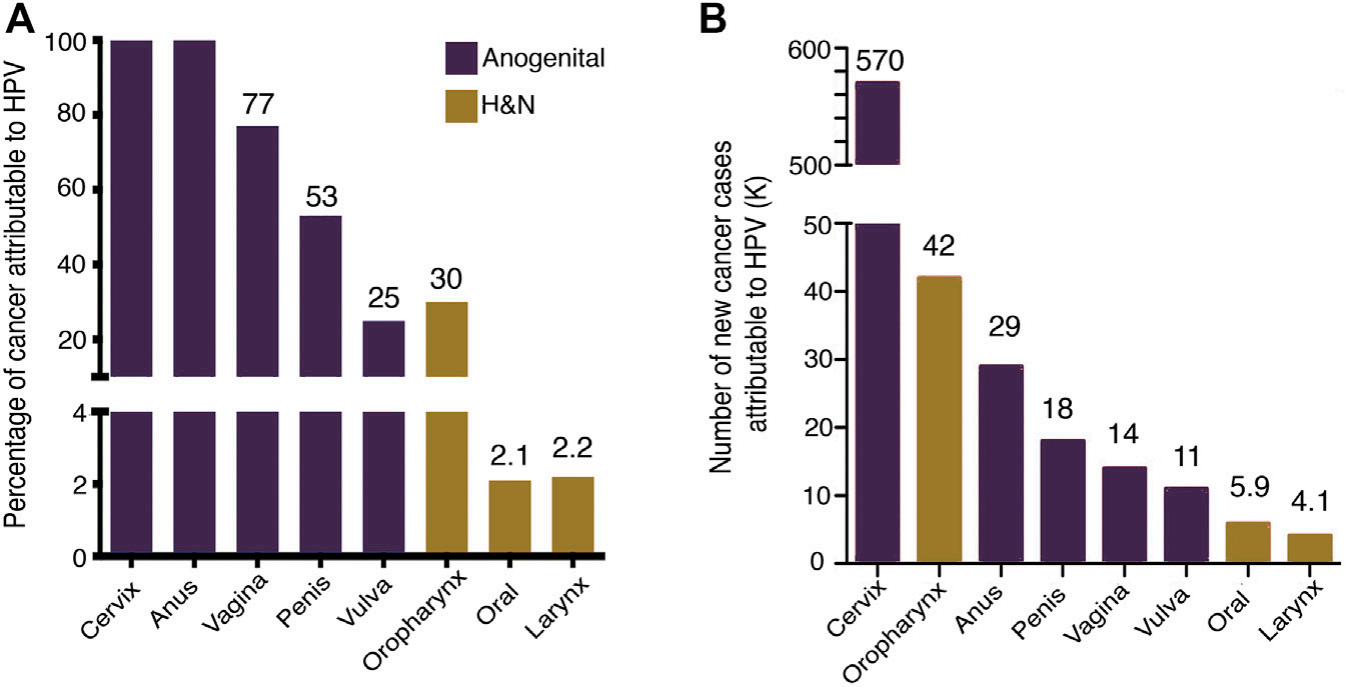
HPV-attributable cancer. **(A)** The graph shows the proportion of cancer attributable to HPV worldwide **(B)** Number of new cancer cases attributable to HPV. Data from de Martel et al. (2020).

In 2020, 19.3 million new cancer cases and 10 million deaths were estimated worldwide (Sung et al., 2021). About 13% of cancer cases are attributed to infections (2.5 million), being Human Papillomavirus (HPV) the second most frequent infectious agent with 31% of cases (de Martel et al., 2020). The HPV-associated neoplasms are the cancer of the cervix, anus, vulva, penis, vagina, and head and neck squamous cell carcinomas (HNSCC). The health problem of HPV-associated neoplasms is concentrated mainly in women since 80% is due to cervical cancer (CC) (de Martel et al., 2020). The main contributions to the knowledge and control of HPV-associated cancer have been developed from CC studies. Persistent high-risk HPV (HR-HPV) infection is responsible for nearly 100% of cervical carcinomas, anal carcinomas, and to a lesser extent HNSCCs (Figure 1).

Recent data estimated that CC alone accounted for more than 600,000 new cases and more than 340,000 deaths (Sung et al., 2021). HPV infection is the most common sexually transmitted infection; it is estimated that at least 80% of people have been infected with HPV before 45 years (Chesson et al., 2014).

HPV belongs to the *Papillomaviridae* family, composed of 52 genera (ICTV 2020). According to the International Human Papillomavirus Reference Center, 222 distinct genotypes are registered (de Villiers et al., 2012). The International Agency for Research on Cancer (IARC) classifies 12 genotypes (16, 18, 31, 33, 35, 35, 39, 45, 45, 51, 52, 56, 58, and 59) as type 1 carcinogens, genotype 68 as a possible carcinogen and several more as probable carcinogens (26, 53, 66, 67, 70, 73, 82, 30, 34, 69, 85, and 9) (IARC 2012). Due to epidemiological and carcinogenic evidence, high-risk genotypes are recognized (the 12 genotypes classified as carcinogens type 1, and the genotype 68), constituting 96% of CC cases (Arbyn et al., 2014).

According to data collected worldwide, the 10 most common genotypes in CC are 16, 18, 45, 33, 58, 52, 31, 35, 39, and 59 (Bruni et al., 2021). While in HNSCC the most frequent HPVs in decreasing order are: 16, 18, 33, 35, 52, 45, 39, 58, 31, 53, and 56 (Tumban 2019). Differences in the frequency of genotypes have been described, which may be related to ethnicity and detection methods. However, because of their high frequency, HPV16 and 18 genotypes have been mostly studied from a functional point of view. A consistent finding is that HPV16 has been the most frequent genotype in cancer at different anatomic sites. The oncogenic potential of HPV16 is associated with persistent infection and a high viral load. Also, different variants of the same genotype possess distinct carcinogenic capabilities.

Recent metagenomics and next-generation sequencing (NGS) studies describe a wide variety of HPV genotypes at various anatomical sites in both healthy tissues and malignant neoplasms. In 69% of healthy subjects, the presence of some HPV genotype has been identified, either in the intestine, mouth, skin, or vagina, being more frequent in the skin (61%) and vagina (41%) (Ma et al., 2014). In anogenital warts, HPV 6, 7, and 11 have been identified as the most frequent genotypes (Xu H et al., 2019). HPV genotypes show greater diversity in low-grade squamous intraepithelial lesions (LSIL) than HSIL, with HPV39 dominating in LSIL and HPV16 in HSIL (Shen-Gunther et al., 2021).

## 2 Structure and Function of Human Papillomavirus Genome

Human papillomaviruses are epitheliotrophic species-specific viruses. HPV viral cycle is closely related to the differentiation of stratified squamous epithelium. About its genomic traits, HPV has a circular double-stranded genome of approximately 8,000 bp. In general, its genomic structure is composed of three regions: the early region (E), which encodes proteins responsible for the regulation of transcription and replication; the late region (L), which encodes structural proteins; and the long control region (LCR) or upstream regulatory region (URR). Transcription is polycistronic as in other viral species. In HPV16, the P97 and P670 promoters are the major transcription regulators. The P97 promoter controls the expression of the E6, E7, E1, E2, E4, and E5 polycistron in undifferentiated cells. The second promoter regulates the transcription of E1^E4, E5, L1, and L2 and is activated in differentiated cells (Wooldridge and Laimins 2008). The general functions and interactions of HPV-encoded proteins are briefly described below.

E1: This viral protein has the largest and most conserved ORF of the papillomavirus. The main function of E1 is to bind to the origin of replication to maintain viral replication and is responsible for maintaining the episomal state. E1 interacts with proteins of the replication machinery (DNA polymerase, RPA, Topoisomerase I and II, PCNA). Interestingly, it also interacts with Histone H1 to displace it from DNA. In addition, E1 has been shown to bind with epigenetic regulators such as HATs, HDACs, and SWI/SNF for chromatin remodeling (Bergvall et al., 2013).

E2: E2 is a key regulatory protein encoded by all papillomaviruses. Interestingly, the short forms of splicing can act as repressors of E2. E2 can interact with specific viral DNA sequences, mainly methylation-dependent sequences, such as E2BS sites located within the LCR region. In addition, E2 is very important for the recruitment of E1 to the replication origin. E2 protein has a repressor role in E6 and E7 transcription, which has been associated with increased apoptosis and decreased cell proliferation (Blachon et al., 2005), in part by the consequent increase of p53, so that the disruption of E2 allows the overexpression of E6 and E7 oncoproteins, which promotes the development of the neoplasm.

On the other hand, E1 and E2 are known to be involved in the process of viral genome insertion into the host genome. However, only the E1 gene is involved in low-grade cervical intraepithelial lesions, and in high-grade lesions, the E1 and E2 genes are also disrupted. The hinge region of the E2 gene has been reported to be unstable making it possibly the most common break when HPV DNA integrates into the host genome (Cricca et al., 2009). Therefore, E2 and E6 have been used as surrogate markers to discriminate CIN2+ in CC (Chen et al., 2020).

E2 functions as a histone code reader by interacting with the bromodomain containing 4 (Brd4) and interacting with acetylated histones in chromatin. E2 has been shown to bind to other chromatin regulators such as HAT (p300) HDAC 1, 2, and 3, KDM5C, SWI/SNF, TIP60, EP400, and SMCX (McBride 2013; Fontan et al., 2020).

E8^E2: E2 splicing isoforms are transcribed from the promoter E8 located in E1. E8^E2 is a DNA-binding protein, which has been characterized as a transcriptional and viral replication repressor through the recruitment of the NCoR platform (Dreer et al., 2016). This isoform participates in the maintenance of the episomal state of the virus and limits viral replication.

E4: E4 localizes to the central region of E2 and is synthesized as a fusion protein of the E1^E4 transcript from promoter 8. E4 is the most abundant protein in the infected epithelium. This protein participates in amplification, release, and transmission of the viral genome. It also promotes the viral productive phase associated with epithelial differentiation-dependent post-translational modifications and participates in cytokeratins collapse and in G2 cell cycle arrest. It has been shown that E4 expression decreases as CIN grades increase until it disappears. E4 also correlates with low levels of methylation in the CADM1, MAL, or miR124-2 genes (Zummeren et al., 2018). These genes are implicated in cervical carcinogenesis and are discussed later in this review. E4 also participates in chromatin regulation by preventing the interaction of MCM2 and MCM7 with chromatin (Wilson et al., 2005; Doorbar 2013).

E5: E5 is mainly localized in the intracellular membranes of the endoplasmic reticulum and Golgi apparatus. In these compartments, it binds with vacuolar ATPase to decrease endosomal acidification, which leads to the degradation of cell surface receptors. Studies have shown that E5 plays an essential role in carcinogenesis, as it is critical in cell transformation and immune modulation by interacting with the major histocompatibility complex class I (MHC-class I). In addition, this protein is involved in several biological processes such as differentiation-dependent viral cycle, DNA synthesis in supra-basal layers, viral amplification, and maintenance of the episomal state, and in the expression of late genes (DiMaio and Petti 2013). E5 activates EGFR in a ligand-independent manner, thus affecting signaling pathways such as MAPK, PI3K-AKT. E5 expression is associated with increased cyclooxygenase 2 (COX2) (Ilahi and Bhatti 2020).

E6 y E7: One of the best described activities by HPV-HR proteins in the transformation process is the inactivation of the tumor suppressor genes p53 and pRB by E6 and E7, respectively (Vats et al., 2021). However, many cellular proteins are affected by direct or indirect interaction with these viral oncoproteins.

E6 is epigenetically regulated by CTCF and can generate distinct isoforms by splicing. Splicing products (E6*) antagonize the effect of full-length E6. E6 participates in immortalization, evasion of apoptosis, evasion of the immune response, the inhibition of the interferon response, participates in epigenetic reprogramming, and regulates chromatin structure through interaction with CBP and P300 (Vande Pol and Klingelhutz 2013; Estevao et al., 2019; Mac and Moody 2020; Scarth et al., 2021; Vats et al., 2021).

E7 is epigenetically regulated by CTCF. It is considered the main protein with transforming activity. It participates in the reprogramming of the cellular environment to facilitate viral replication and reduces the expression of MHC I in conjunction with E5. Like E6, E7 interacts with multiple proteins to regulate epigenetic mechanisms. E7 has been shown to interact with and induce the activity of DNMT1, DNMT3a, EZH2, CBP, and P300. In addition, E7 modulates viral replication by interacting with HDAC1 and HDAC2 *via* Mi2β. E7 induces KDM6A and KDM6B expression, leading to host cell reprogramming by re-activation of HOX and p16INK4A genes. E7 also post-transcriptionally increases SETD2 levels and regulates H3K36me3 marks on viral chromatin (Burgers et al., 2007; Roman and Munger 2013; Saha et al., 2017; Gautam et al., 2018; Pal and Kundu 2019; Mac and Moody 2020; Vats et al., 2021).

Studies show that enhancer of zeste homolog 2 (EZH2) is specifically activated by E7 from HPV16 through the release of E2F. EZH2 is an enzymatic subunit that mediates H3K27me3 and contributes to cell proliferation (G1-S) involving several mechanisms such as the stimulation of Cyclin D1 and E, as well as retinoic acid signaling or the WNT pathway. In addition, EZH2 can suppress apoptosis through the silencing of the miRNA-31 promoter, which has among its regulatory targets the antiapoptotic gene E2F6 (Holland et al., 2008; Zhang et al., 2014).

Despite EZH2 increase by E6 and E7, the H3K27me3 mark does not increase; this is associated with the Ser21 phosphorylation, which is mediated by AKT. This modification prevents the methylation enzymatic activity while it increases the expression of histone demethylase KDM6A, associated with activation of HOX genes (Hyland et al., 2011).

Late ORF: L1 is the major capsid protein, which, can self-assemble into virus-like particles (VLP) and is mainly involved in the cell infection process. The L1 region of HPV is susceptible to DNA methylation which, interestingly, has been associated with lesion grade and viral integration. For example, methylation of CpG 5698 and 5617 is associated with high-grade lesions and CC (Turan et al., 2006; Buck et al., 2013; Wang and Roden 2013; Torres-Rojas et al., 2018).

LCR: Regarding the LCR region, it is a viral sequence that does not encode proteins, but it is one of the most important regions of the virus since it contains the origin of replication and interacts with multiple epigenetic regulators and transcription factors. Comprising approximately 10% of the genome (Bernard 2013).

## 3 Changes in the Human Papillomavirus Epigenome

Human papillomavirus DNA associates with proteins similar to H2A, H2B, H3, and H4 histones. Electron microscopy analyses revealed that the complete HPV genome contains 32 nucleosomes, each measuring about 12 nm in diameter (Stunkel and Bernard 1999; Burley et al., 2020). Interestingly, HPV chromatin is also epigenetically regulated through various histone post-translational modifications (acetylation, ubiquitination, methylation, and phosphorylation among others) and viral DNA methylation (Mac and Moody 2020).

Changes in chromatin are closely linked to the epithelial differentiation process. Viral promoters contact each other by three-dimensional folding of nucleosomes at different stages of the viral cycle. Some authors have proposed that chromatin modifications occur mainly in the proximal upstream regulatory region of E6 in undifferentiated cells, whereas in differentiated cells, it occurs in the late promoter region (del Mar Pena and Laimins 2001). However, some studies show that late promoters are not fully repressed in basal cells (Wooldridge and Laimins 2008).

Initially, when HPV infects undifferentiated cells of the basal layer of the epithelium, the viruses remain in an epigenetically repressed episomal state, with enrichment of H3K27Me3 and low levels of H3K4Me3. Consequently, there is a low viral load (between 50 and 100 viral copies per cell), which favors a deficient activation of the immune response (Ferguson et al., 2021).

Chromatin immunoprecipitation assays on HPV31 revealed transcriptionally active chromatin marks such as acetylated H3K4me2 in HPV promoter regions p97 (early in LCR) and p742 (late in E7). Interestingly, these epigenetic marks increase after differentiation triggering E6/E7 transcription. In contrast, undifferentiated cells carry repressive marks such as H3K9me2 and H3K27me2 (Wooldridge and Laimins 2008).

Notably, the p97 promoter is regulated from the 5′ end in the LCR region; therein lies the origin of replication, the keratinocyte early enhancer (KE) and an auxiliary enhancer (AE) that enhances KE activation (Kanaya et al., 1997).

**FIGURE 2:**
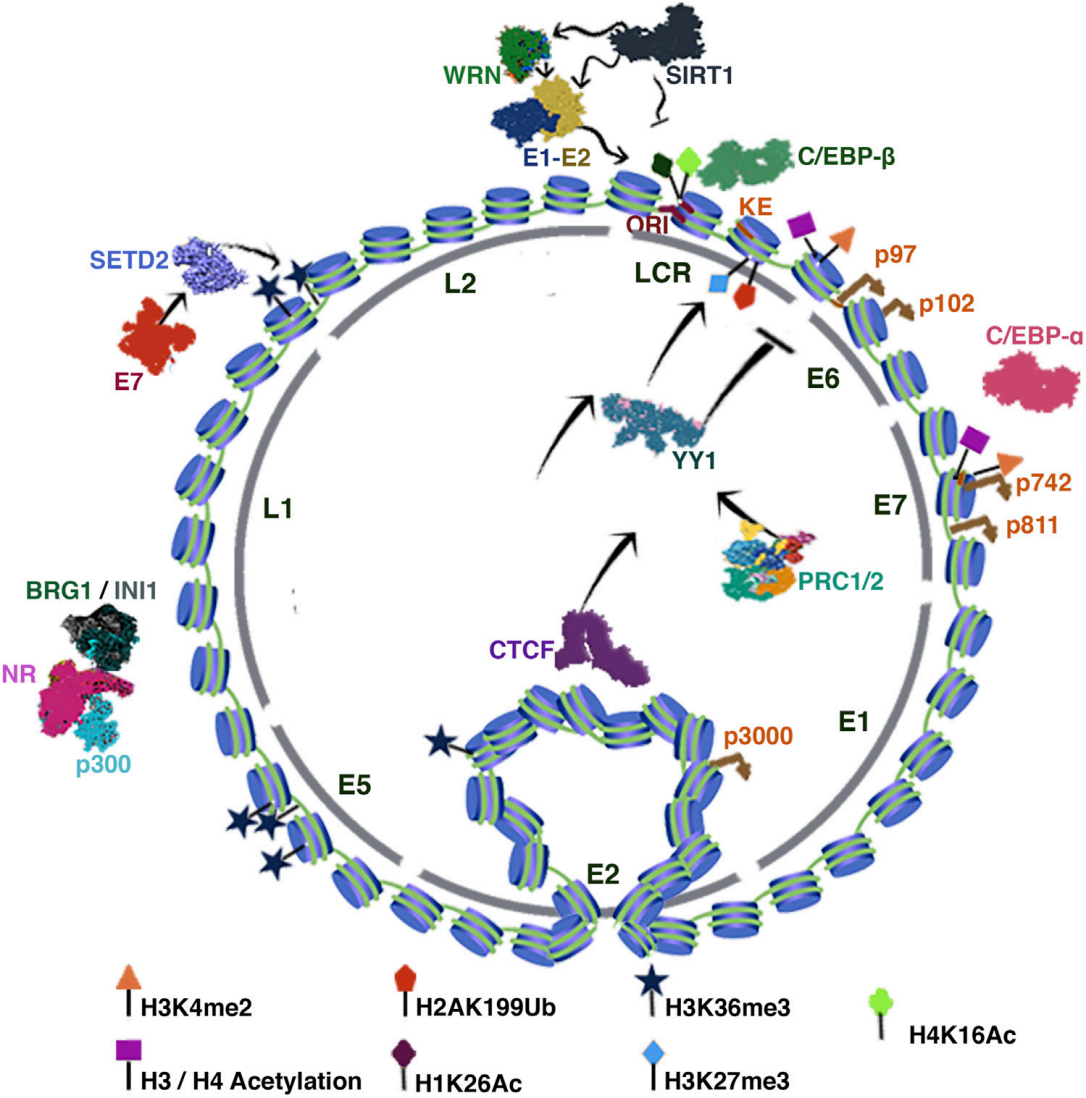
HPV Chromatin. The figure illustrates the HPV chromatin regulation. The inner-circle indicates the distribution of the viral genes. The tags indicate the position of the corresponding chromatin mark. In addition, the characteristic loop between CTCF and the LCR region is illustrated. See text for more detail.

Transcriptional activation marks recruit factors such as C/EBP-β and C/EBP-α to the LCR and late promoter sequences. Notably, the KE region is also an enhancer of the late promoter, so it has been proposed that differentiation alone might be enough to mildly induce late transcription without active viral replication (Wooldridge and Laimins 2008) (Figure 2).

Some studies have shown that negative regulation of the HPV-16/18 E6-E7 promoter requires the binding of YY1 to the promoter KE sequence (Lace et al., 2009). YY1-directed epigenetic silencing involves the recruitment of Polycomb repressor complexes 1 and 2 (PRC1 and PRC2) to viral chromatin leading to the enrichment of H3K27me3 and H2AK199Ub marks. Interestingly, epithelial differentiation inhibits YY1 expression, resulting in positive regulation of E6 and E7 (Mac and Moody 2020). Notably, YY1 regulates HPV 16, 18, 11, and 8 promoter sequences; however, the mechanism of action has not been fully elucidated (Lace et al., 2009).

HPV infection is initially established in undifferentiated basal cells, so there is no expression of E6 and E7. This transcriptional state is also dependent on CCCTC binding factor (CTCF). Studies in HPV 18 have described that CTCF can interact with the E2 ORF and, in turn, with YY1, whereby CTCF-YY1 mediates a three-dimensional interaction between the E2 ORF and the LCR region to establish a repressive chromatin loop (Pentland et al., 2018). Epithelial differentiation disrupts the CTCF-dependent chromatin loop (Figure 2). Studies in HPV18 showed that abrogation of CTCF binding to the E2 ORF increases the transcriptional activity of the p102 early promoter, increasing the expression of E6/E7 proteins (Ferguson et al., 2021).

Recent findings have shown that viral integration events frequently trigger host chromatin changes, which enhance the carcinogenic process in HPV+ tumors. Interestingly, CTCF is a key regulator in these processes, since mutations that disrupt the binding sites as well as the introduction of new sites for CTCF can reorganize chromatin and modify transcriptional regulation, allowing tumorigenesis beyond the transcriptional regulation of E6/E7 (Karimzadeh et al., 2022). In addition, sequencing studies elucidated that CTCF may play an important role in the regulation of alternative splicing of viral genes (Ferguson et al., 2021).

Studies using C33a cells infected with HPV16 have revealed that the histone deacetylase sirtuin 1 (SIRT1) is recruited to the HPV replication origin to become part of the functional E1-E2 complex. Loss-of-function assays of SIRT1 by CRISPR-Cas9 showed increased replication of E1-E2 due to a growing stability of E2 caused by an increase in acetylation (Das et al., 2017). Interestingly, Dipon Das et al. showed that SIRT1 post-transcriptionally regulates the DNA repair protein Werner helicase (WRN) recruited to the E1-E2 complex to regulate replication fidelity. Like SIRT1, deletion of WRN also results in uncontrolled viral replication (Das et al., 2019) (Figure 2). By chromatin immunoprecipitation (ChIP) assays, SIRT1 was found to bind to multiple regions of the HPV31 genome except for the L1 region. Particularly in undifferentiated cells, SIRT1 binds to the LCR, erasing the H1K26Ac and H4K16Ac acetylation marks, thus repressing HPV31 late gene expression (Langsfeld et al., 2015) (Figure 2).

Interestingly, HPV31 E7 has been reported to increase SETD2 protein levels. SETD2 is a histone methyltransferase that writes the H3K36me3 mark (active transcription) on viral chromatin. SETD2 can collaborate in the viral cycle regulation by recruiting of H3K36me3 readers to HPV. Furthermore, experiments in CIN612 cells showed that SETD2 writes the H3K36me3 mark abundantly in E2, E4, and E5 ORFs and not in E6 and E7 (Figure 2) (Gautam et al., 2018).

Likewise, the nuclear receptors have an essential role in viral chromatin regulation since they allow the recruitment of chromatin remodelers such as BRG1 and INI1 (Figure 2). These proteins relate to SWI/SNF complex that can recruit the MLL1 and SETD1A enzymes to write the H3K4me1/3 marks, these complexes can also recruit HAT proteins such as p300 (Groves et al., 2016).

### 3.1 Viral DNA Methylation

**FIGURE 3:**
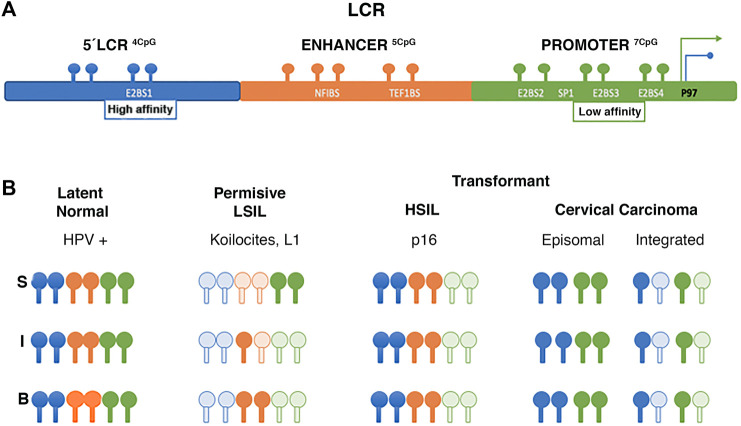
Changes of methylation in LCR of HPV 16. **(A)** LCR region binding sites. **(B)** Schematic illustrating LCR methylation changes of HPV 16 isolated from different epithelial layers. The letters “S,” “I,” and “B” refer to superficial, intermediate, and basal layers respectively, and at different stages of viral infection and transformation. Dark spots show methylated CpG clear spots show unmethylated CpG. Data from Vinokurova and von Knebel Doeberitz 2011, Chaiwongkot et al. (2013).

The first study to analyze viral DNA methylation, reported changes in the LCR with potential gene regulatory implications (Burnett and Sleeman 1984). Subsequent studies determined that these methylation changes are associated with the viral life cycle and the cell differentiation. The upstream regions of LCR (3′of L1) and E6 have been of particular interest in some methylation studies (Johannsen and Lambert 2013). Vinokurova and von Knebel Doeberitz (2011) determined CpG methylation changes on the LCR of HPV16 isolated from epithelia by microdissection. In epithelium lacking cytopathic alterations, CpGs remain methylated, whereas, in low-grade lesions or permissive infections, changes in methylation are related to the epithelial differentiation layer, keeping the E2-binding site (E2BS) of the promoter region demethylated. In contrast, E2BS1 is methylated in high-grade lesions and is associated with early promoter activation (P97) and viral integration (Vinokurova and von Knebel Doeberitz 2011). Regarding invasive carcinoma, Chaiwongkot et al. (2013) identified differences in methylation as a function of viral integration status, specifically at the E2SB1 (7453 and 7459), E2SB3 (37, 43) and E2SB4 (52, 58) sites of HPV16 LCR; methylation being lower in carcinomas with the integrated virus than in episomal carcinomas (Figure 3). In addition, viral genome copy number appears to be related to methylation status, as higher methylation levels were identified at LCR in cases with higher integrated copy number (Chaiwongkot et al., 2013).

Piyathilake et al. (2014) reported an association between low risk CIN2+, and high methylation levels in the HPV 16 promoter and enhancer regions. Additionally, women with high folate concentrations and high methylation have a lower risk of being diagnosed with CIN2+. A similar effect was observed with vitamin B12, suggesting that folate and vitamin B12 intake may reduce HPV hypomethylation associated with the risk of developing CIN2. That is, two of the most important methyl donor micronutrients are associated with site-specific methylation of the promoter (position 31, 37, 43, 43, 52, and 58) and enhancer (7862) regions of the E6 gene, which is associated with a low probability of CIN2+ diagnosis (Piyathilake et al., 2014) (Figure 3).

Several authors have reported increased methylation in the L1 region associated with CIN3 or CC. Recently Bee et al. identified that methylation of the CpG 5615 site of L1 of HPV52 is most frequently associated with NIC3, and SNPs associated with the C clade (Bee et al., 2021). Additionally, increased DNA methylation has been observed in the L1, L2, E2, and E5 regions in high-grade lesions. As the degree of lesion progresses grade advances, increased methylation at L1 is more evident in HPV16 than in HPV18 (Torres-Rojas et al., 2018). Concerning viral integration status, it is more frequent in HPV18 cases than in HPV16 (Torres-Rojas et al., 2018). These data indicate that the viral methylation/integration profile may be related to genotype and viral species. In this regard, in cervical cancer the 3′ LCR methylation and E1/E2 region integrity are higher in HPV16 than in HPV18 and HPV45 (Amaro-Filho et al., 2018). It has been shown that viral integration is dispensable for malignant transformation and the alternative mechanism for E6 and E7 deregulation could be DNA methylation at the E2SB sites in HPV16 (Cheung et al., 2013).

## 4 Epigenetic Alterations in Human Papillomavirus-Associated Neoplasms

Diverse evidence has shown that patients with HNSCC may have different responses to treatment depending on HPV status, mainly due to transcriptomic changes involving genes related direct or indirectly by viral presence (Pyeon et al., 2007; Lohavanichbutr et al., 2009; Mendez-Matias et al., 2021).

The characterization of epigenetic alterations in neoplastic transformation is a significant event. Unlike genetic alterations, epigenetic changes are potentially reversible. Although the mechanisms that lead to epigenetic alterations are not fully understood, it is relevant that they could be used as diagnostic and prognostic biomarkers as well as therapeutic targets. This section will focus on some of the epigenetic alterations in precursor lesions and invasive cancer and their potential use as biomarkers.

Sartor et al. (2011), identified by global methylation analysis that HPV-positive cell lines have higher levels of methylation in LINE-1 genic regions compared to negative ones, 77% and 45%, respectively. It was reported that the 19q13 region has high methylation rates; as well as some target genes of the polycomb complex (CCNA1, PREX1, RUNX2, and SPON2). Several differentially methylated regions (DMR) have been found in tumors, as well as in genes of great biological importance such as ESR1, and DCC (important in HNSCC) (Sartor et al., 2011).

Lechner et al. (2013), identified an HPV + HNSCC-associated methylation signature, regardless of anatomic site (Lechner et al., 2013). Additionally, two HPV+ subgroups were identified, associated with high levels of methylation and poor prognosis. Other studies have also identified two subgroups within HPV+ based on expression and viral integration status (Ren et al., 2020).

In contrast, Brennan et al. characterized five subtypes of HNSCC based on methylation profiles, one of them associated with HPV that corresponds to the previously described atypical expression profile and is associated with better survival (Brennan et al., 2017). Nakawaga et al. (2020), identified four subtypes of HNSCC; two of them are associated with HPV, another presents high levels of methylation in promoter regions and correlates with a better prognosis, and the last one has intermediate levels of methylation (Nakagawa et al., 2020).

Ren et al. (2018), identified a set of 17 DMRs in OPSCC-HPV+, however, only seven regions are capable of discriminating HPV+ OPSCC from healthy tissue (KCNA3, EMBP1, CCDC181, ELMO1, C1QL3, MIR129–2, and ZNF137P) (Ren et al., 2018).

Misawa et al. (2020), identified that methylation of 10 genes is associated with recurrence in OPSCC HPV+, this signature includes ATP2A1, MRGPRF, GNMT, GPT, LYNX1, MAL, MGC16275, CALML5, DNAJC5G, and LY6D, being the last three from circulating tumor DNA (Misawa et al., 2020).

Recently, Berglund et al. (2022), identified DMRs in HNSCC HPV16; the most significant were: SYCP2, MSX2, HLTF, PITX2 and GRAMD4. Interestingly, the methylation profiles with genotypes of the A7 species such as HPV 31, 33 and 35 are compatible with those presented in HPV16, and a different methylation profile was found in cases with genotypes belonging to other viral species (Berglund et al., 2022).

Comparing with normal tissue, 86 DMRs were identified in anal carcinoma and 36 in CC, of which 17 DMRs are shared in both malignancies (ASCL1, ATP10A, CCDC81, DPP10, FMN2, MARCH11, MIR129-2, PAX1, PEX5L, RYR2, SORCS3, T, WDR17, ZIK1, ZNF154, ZNF177, ZNF529/ZNF382) (Siegel et al., 2021).

Alternatively, different DMRs and differentially hydroxymethylated regions (DhMR) in genes involved in the Hippo pathway were identified in NIC 3 and CC. Interestingly, the number of DMR increases as the degree of injury progresses; however, the DhMRs decrease from NIC 3 to CC.

Some genes from the DMRs were associated with survival (DES, MAL, MTIF2, PIP5K1A, RPS6KA6, ANGEL2, MPP, and PAPSS2) (Han et al., 2021). Gagliardi et al. (2020) identified a greater number of DMRs in cervical tumors with genotypes of the A9 vs. A7 species. In addition, tumors with A7 genotypes have higher H3K4me1 (associated with enhancers) and H3K27Ac (associated with active promoters), while tumors infected with genotypes of the A9 species have an increase in H3K4me3 (associated with active promoters). This could have implications in the expression profiles and tumor aggressiveness (Gagliardi et al., 2020).

Globally, a greater number of methylated regions have been reported in HPV-positive cell lines and malignancies than in negative ones (Sartor et al., 2011; Ren et al., 2018). However, in HPV-positive squamous cell carcinoma of the penis (PeSCC), it was reported that 77% of the variable methylation positions (MVPs) are hypomethylated (Feber et al., 2015). Among the 960 MVPs associated with HPV, there are WNT pathway genes such as GRAMD4 and GPX5.

It is noteworthy that 30 MVPs were validated in independent cohorts in HNSCC and CC, managing to stratify the HPV+ cases from the negative ones according to epigenetic signature; additionally, significant differences were shown in survival at 5 years, in HNSCC (81% vs. 38%) and in CCSCC (77% and 50%), which was a better predictor of survival than the sole presence of HPV (Feber et al., 2015).

In other PeSCC studies, 65 differentially methylated genes were identified, with inverse correlation with expression in HPV+ cases. Among which are CD70, HN1, FZD5, FSCN1, and PRR16 (Kuasne et al., 2015).

The 5-hydroxymethylation of the cytokine (5hmC) associated with loss of transcriptional repression has been identified in lower levels in HPV + HNSCC compared to HPV- cases. Among the genes associated with hyper-5hmC in HPV+ tumors are CDKN2A and enrichment of pathway genes such as desmosomes, NF-kB, oxidoreductase activity, and mesenchymal cell differentiation where genes such as SNAI2, BMP2, SMAD2 and TGFB2 are found (Liu et al., 2020).

Many cellular genes have been proposed as diagnostic and prognostic biomarkers in CC and its precursor lesions (Hesselink et al., 2014; Kalantari et al., 2014; Verhoef et al., 2014; Boers et al., 2016). Global methylation profiles analysis in 12 cancer types identified 4 CC-specific markers cg07211381 (RAB3C), cg12205729 (GABRA2), cg20708961 (ZNF257), and cg26490054 (SLC5A8) with 96.2% sensitivity and 95.2% specificity. Additionally, this methylation profile can distinguish between tumors and normal tissues (Xu W et al., 2019).

Nowadays, the methylome offers several candidates with potential as clinical biomarkers and therapeutic targets in HPV+ neoplasms. In addition, non-coding RNAs have been highlighted in the scientific and therapeutic landscape. Interestingly, some ncRNAs are transcribed by the own HPV.

## 5 The Pathogenetic Role of ncRNAs in HPV+ Cancers

The invention of new sequencing technologies, bioinformatics tools, as well as new experimental strategies, have made possible the discovery of new functional molecules of non-coding RNA (ncRNA) (Panda 2018; Huang et al., 2019). It is well known that HPV can transcribe non-coding RNAs such as circular RNAs (circRNAs) and microRNAs (miRNAs). Furthermore, it has been suggested that the altered expression of some long-noncoding RNAs (lncRNAs) in some types of cancer is a direct consequence of the expression of the viral oncoproteins E6 or E7. Therefore, we performed a bibliographic search for ncRNAs encoded directly by HPV.

In this section, we begin describing the HPV non-coding transcriptome, and finally, we highlight the function of lncRNAs induced by the viral oncoproteins in the host cell.

### 5.1 Human Papillomavirus Non-coding Transcriptome

**FIGURE 4:**
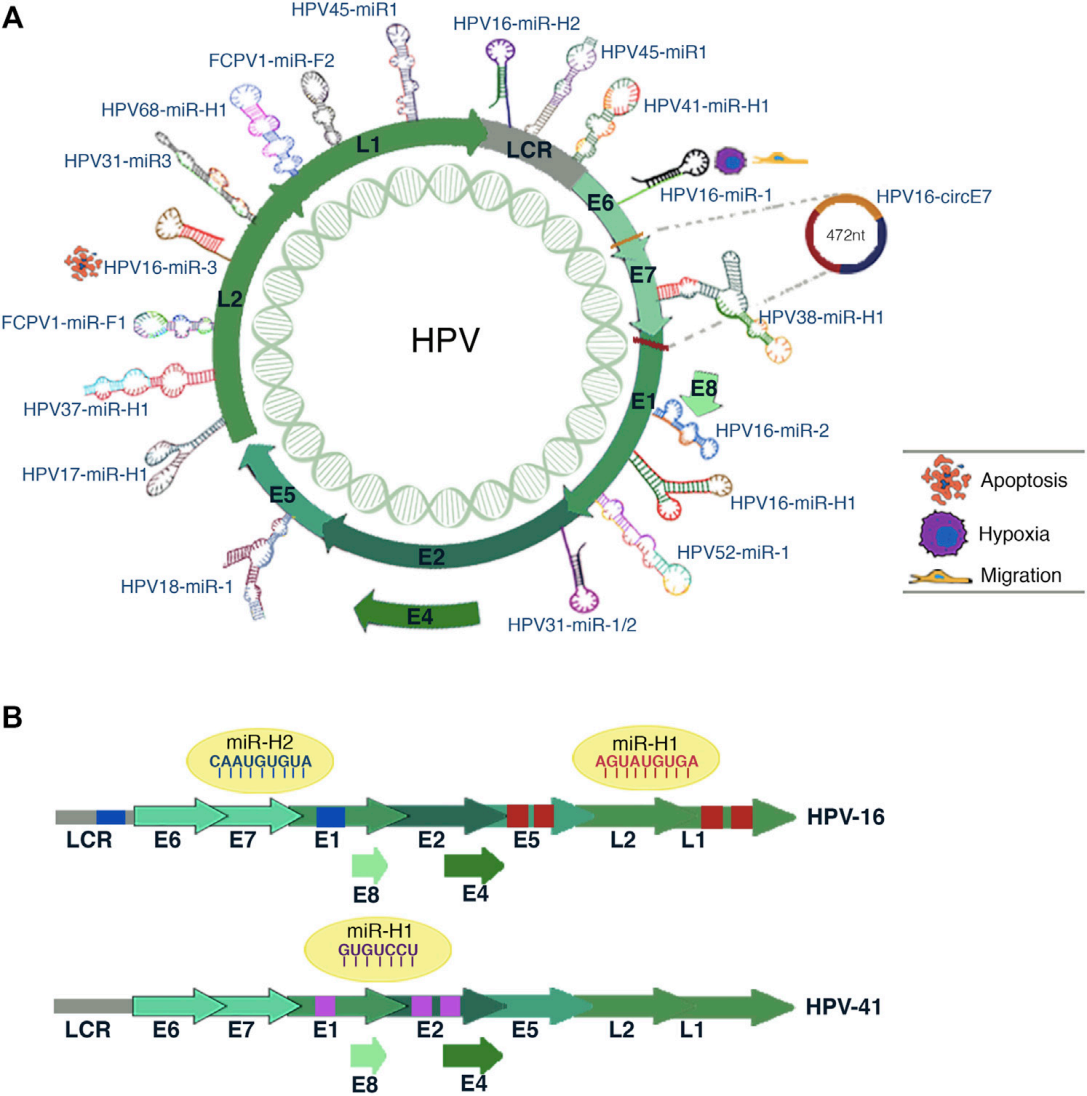
HPV-encoded miRNAs. **(A)** Illustration shows the miRNAs coding site inside the genome of different HPV subtypes. The predicted biological processes for some HPV16 miRNAs are depicted in the figure. The circE7 synthesis site is also shown. **(B)** The scheme represents the potential interaction sites of miRNAs in the viral genome of HPV-16 and HPV-41. The predicted target viral sequences location for each miRNA are shown in the same color as the corresponding miRNA sequence (blue, red, or purple).

Recent studies have shown that within the HPV genome, there are regions that contain circRNA and miRNA genes (Figure 4).

CircRNAs are single stranded, covalently closed, circular RNA molecules. CircRNAs can be generated from several genomic regions (intergenic, intronic, and coding). Regarding the biosynthesis of circRNAs, two biosynthesis models have been proposed, and both involve back-splicing (Miao et al., 2021).

Although they have been little studied, there is evidence showing that they perform essential cellular functions. These molecules can act as miRNA sponges, or in some cases, they might indeed have coding potential, as is the case of circE7 (Panda 2018). HPV16 cervical carcinoma cells have high expression levels of circE7 (Zhao et al., 2019) (Figure 4A), and this circRNA is primarily localized in the cytoplasm and requires post-transcriptional modification of N6-methyladenosine (m6A) in the UTR to associate with polysomes and enhance E7 protein levels thus promoting cancer cell growth. These new findings show how these types of nucleic acids transcribed from HPV aid in malignant transformation and increase oncogenic potential in cells.

There are few published studies regarding the miRNAs transcribed from the HPV genome. However, it has been shown that viral miRNAs modulate cellular and viral gene expression, promoting carcinogenesis and evading the host’s immune system. Shun-Long Weng et al. (2018) performed bioinformatic analyzes to identify HPV-encoded miRNAs. They analyzed genomes from different high-risk HPV genotypes to identify viral pre-miRNAs based on phylogenetic information and structural evolution. Notably, HPV16 showed coding potential for HPV16-miR-1, HPV16-miR-2, and HPV16-miR-3, which are located inside the ORFs of E6, E1, and L2, respectively (Weng et al., 2018) (Figure 4A). The authors also identified possible targets of these miRNAs; however, these findings require experimental validations.

The predicted targets of HPV16-miR-1 (ARID5B, ZEB2, THBS1, and STAT5B) are mainly involved with biological processes such as cell migration, cell motility, response to hypoxia, and regulation of cell adhesion. For HPV16-miR-3, the predicted targets (SYNE1, GATA6, GULP1, PDE1B, IGFBP4, PRELP, and MYH1) are associated with cell death, development, and cell differentiation (Figure 4A). Regarding HPV16-miR-2, the predicted targets are AFF3, FRMD7, IGDCC4, MYRIP, NRN1, PMP22, and RBPMS; however, these genes do not associate with cervical cancer progression (Weng et al., 2018). Strikingly, some of these miRNAs could be targets for new antiviral drugs, and miRNAs specific to certain HPV subtypes could serve as possible biomarkers. In addition, the authors found putative miRNAs in other HPV genotypes such as HPV-18 (1 miRNA), HPV-31 (3 miRNAs), HPV-45 (2 miRNAs), and HPV-52 (1 miRNA) (Weng et al., 2018) (Figure 4A).

Through sequencing analysis, Qian et al. (2013) discovered nine HPV-transcribed miRNAs. These miRNAs were successfully validated by qPCR and *in situ* hybridization in cervical tissue samples and cervical cell lines (Qian et al., 2013). Two of the validated miRNAs were encoded by HPV 16 (HPV16-mir-H1 and HPV16-mir-H2), one by HPV 38 (HPV38-mir-H1), and one by HPV 68 (HPV68-mir-H1) (Figure 4A).

The authors also found both human and viral target genes of miRNAs encoded by HPV16. HPV16-miR H1 and miR H2 have unique targets in the human genome, and 15 shared target genes (CDC2L6, EIF2C1, IMPAD1, BNC2, SNX27, TNRC6B, BACH2, CYP26B1, DDX19B, FGF7, PBRM1, PHACTR2, RBM3, RGS7BP, TEAD1). Regarding the HPV16 viral genome, authors found four target sequences for HPV16-miR-H1-1, two in the E5 region and two in the L1 gene (Qian et al., 2013) (Figure 4B).

HPV16-miR-H2-1 presented two targets in the viral genome, one in the LCR region and the other in the L1 gene (Figure 4B). A gene ontology analysis suggests that HPV16-miR-H1-1 is involved in processes related to the M phase of the cell cycle, immune system development, adhesion, and migration. These processes are crucial for viral infection, activation of immune cells, and other mechanisms crucial for neoplastic development (Qian et al., 2013; Gallo et al., 2020).

Subsequent studies conducted by Elina Virtanen et al. (2016) evaluated the expression of some miRNAs encoded by HPV 16 reported by Qian et al. (2013). The authors extracted RNA from HPV 16 samples obtained from paraffin-embedded cervical tissue, cell lines, liquid cytology, and cervical cells obtained from colposcopy. Most HPV-encoded microRNAs were detected in all samples; however, HPV16-miR-H2 was not detected in these assays (Virtanen et al., 2016).

Bioinformatic analyzes and multiple experimental strategies performed by Chirayil et al. (2018) showed the potential of various HPV genotypes to transcribe miRNAs. The most significant miRNAs are transcribed from HPV41 (hpv41-miR-H1), HPV17 (hpv17-miR-H1), HPV37 (pv37-miR-H1) and even an HPV from the *Fringilla coelebs* FcPV1 (fcpv1-miR-F1 and fcpv1-miR-F2) (Figures 4A,B). Interestingly, the authors suggested that FcPV and HPV41 miRNAs may regulate viral gene expression as they found potential target sequences for these miRNAs in HPV (Chirayil et al., 2018).

The research field on miRNAs and cirRNAs encoded from HPV encourages new therapeutic strategies based on RNA for various medical applications. However, it would be essential to advance in greater depth in knowing the biological functions of these new molecules.

### 5.2 Long Non-Coding RNAs Regulated by E6/E7 Oncoproteins

LncRNAs are transcripts of more than 200 nucleotides long, which lack coding potential. LncRNAs have been classified in several ways, for example, according to their position concerning neighboring encoding genes (divergent, convergent, intergenic, overlapping—sense/antisense, intronic, among others) (Schmitz et al., 2016) or according to their cis or trans transcription site (Tong and Yin 2021). The diversity of interactions that lncRNAs have with cellular components (RNA-DNA, RNA- Protein, RNA-RNA), have function to classified lncRNAs according to their function as decoys, scaffolds, enhancers, guides, sponges, or competing endogenous (Guttman and Rinn 2012; Castro-Oropeza et al., 2018).

LncRNAs acting as Decoys, induce or prevent proper regulation of gene expression through association with DNA-binding proteins. LncRNA scaffolds assemble various structural complexes to finally adapt them to specific transcriptional regulatory regions (St Laurent et al., 2015). LncRNAs with guide function associates with proteins to direct them to specific regions of the genome and thus cause an effect at the transcriptional or chromatin level. LncRNA enhancers can organize the nuclear architecture to form enhancer contacts thus promoting gene expression. Sponge refers to lncRNAs containing responsive elements to miRNAs, to sequester them and thus prevent their function on their target mRNAs. On the other hand, lncRNAs can act as competing endogenous RNAs, and bind to an mRNA to give it stability by recruiting proteins, thus preventing degradation (Rinn and Chang 2012). Generally, the function of lncRNAs is associated with their subcellular localization, some may regulate specific functions in the nucleus, while others are exported to the cytoplasm, where they exert a different function. The lncRNAs located in the nucleus can modify chromatin by binding epigenetic factors to DNA to regulate transcription and RNA processing. Gene expression regulation is an intricated process that involves epigenetic modifications such as DNA methylation, histone modifications, chromatin remodeling, and ncRNA-based mechanisms. Although the role of nuclear lncRNAs in epigenetic regulation has been extensively investigated, there is little information about the nuclear lncRNAs involved in the epigenetic regulation of HPV-associated neoplasms.

Studies have shown that the presence of HPV in cancerous tissues stimulates the differential expression of lncRNAs. For example, Ma et al. found 107 differentially expressed lncRNAs when comparing normal tissue vs. HPV-negative HNSCC tumors. On the other hand, in the comparision of normal tissue vs. HPV-positive HNSCC, the number of lncRNAs with differential expression increased to 801 transcripts (Ma et al., 2017). Furthermore, studies by Yang et al. compared the transcriptome of HPV-positive cervix cancer cell lines (SiHa and HeLa) vs. an HPV-negative cell line (C33). Interestingly, the authors found changes in the expression of lncRNAs and mRNAs, suggesting that the altered transcripts could be related to HPV-induced proliferation (Yang et al., 2016). These data suggest that HPV involvement is significant in altering lncRNAs in cells.

It should be noted that some studies have proposed lncRNAs expression signatures in cancer associated with HPV infection. However, the molecular mechanisms of action of these molecules have not yet been elucidated. Also, it is still unknown if these expression changes are an indirect consequence of viral infection or if ncRNAs and HPV oncoproteins directly regulate them.

Viral proteins can alter the biological activities of lncRNAs either by binding to them directly or indirectly, thus changing their ability to interact with proteins or other nucleic acids (Sharma and Munger 2020c). HPV, like other viruses, is capable of dramatically altering the expression profile of host cell lncRNAs. Several investigations have identified numerous lncRNAs whose expression is modulated by HPV infection primarily through E6 and E7 proteins (Sharma and Munger 2020c).

**FIGURE 5:**
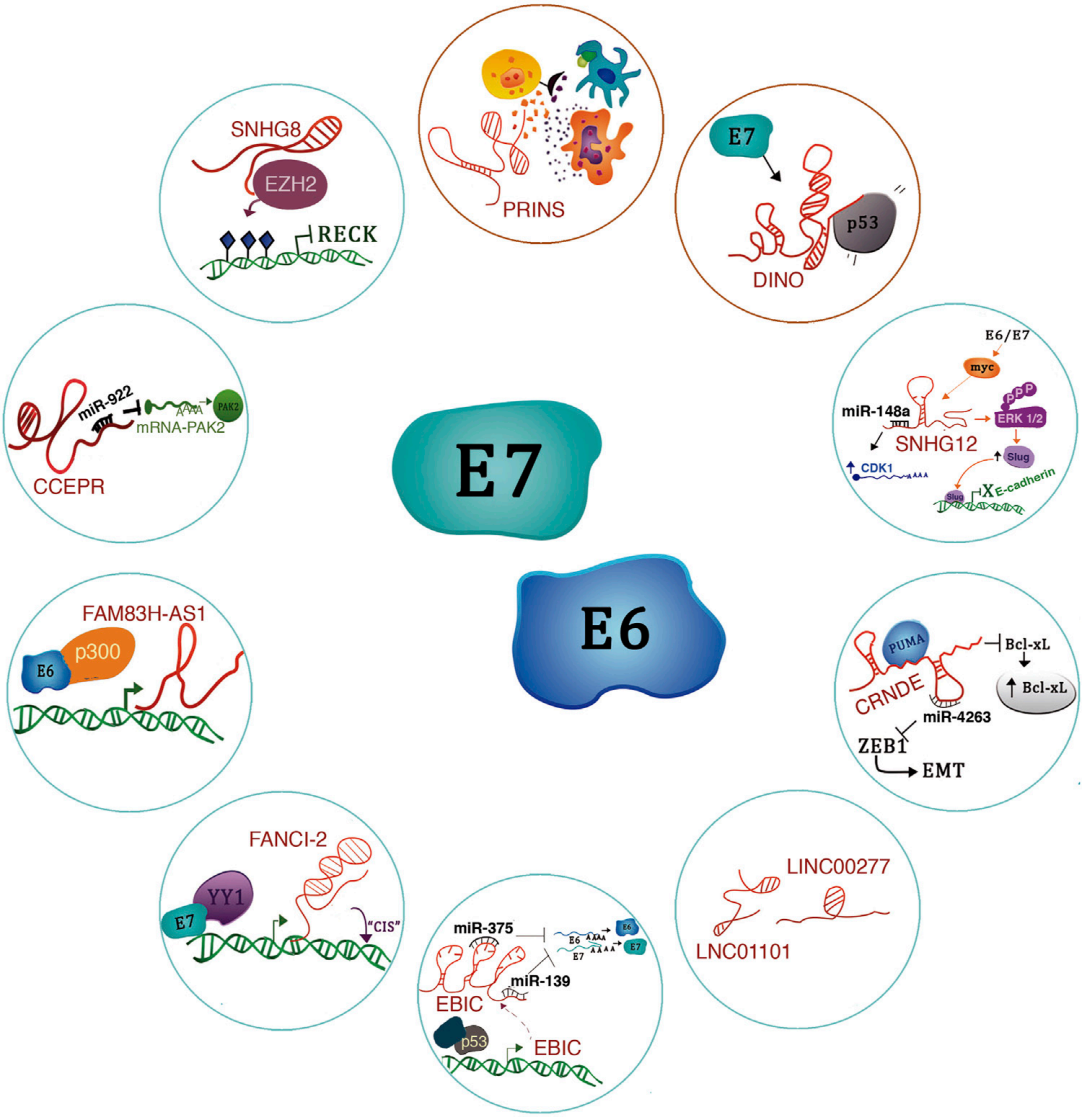
lncRNAs regulated by E6/E7 oncoproteins. The figure shows the molecular mechanisms of lncRNAs regulated by the E6/E7 oncoproteins. Blue circles refer to oncogenic lncRNAs and red circles to tumor suppressors.

Interestingly, lncRNAs have been found to enhance virus invasion and host cell reprogramming to allow sustained infection. Conversely, there are also antiviral lncRNAs that regulate the innate and adaptive immune response to eliminate viral particles. In the following section, we will detail some oncogenic and tumor suppressor lncRNAs that are regulated by E6 or E7 and that influence some features leading to malignancy (Figure 5) (Ouyang et al., 2016).

#### 5.2.1 Oncogenic Long Non-Coding RNAs

lncRNA SNHG8 -A recent study revealed that lncRNA SNHG8 is highly expressed in HPV-induced cervical cancer cells compared to HPV-negative cells (Qu et al., 2020). Notably, this lncRNA has molecular functions in other types of cancer. For example, it acts as a miR-634 sponge in breast cancer, enabling the expression of the ZBTB20 oncogene, thus triggering breast cancer progression (Xu et al., 2021). Also, in hepatocellular carcinoma, the lncRNA SNHG8 acts as a sponge for the tumor suppressor miR-1493 (Dong et al., 2018). Interestingly, in cervical cancer (HPV+), the lncRNA SNHG8 has an epigenetic mechanism. It is mainly located in the nucleus and interacts with EZH2 to promote H3K27me3 enrichment on the RECK (Reversion inducing cysteine-rich protein with Kazal motifs) promoter, thus suppressing RECK expression. Therefore, the functional implications of lncRNA SNHG8 expression include proliferation, migration, and apoptosis inhibition in cervical cancer cells (Figure 5) (Qu et al., 2020).

lncRNA CCEPR -The Cervical Carcinoma Expressed PCNA Regulatory LncRNA (CCEPR) is highly expressed in cervical cancer (Sharma and Munger 2018). For this reason, CCEPR is also known as cervical carcinoma high-expressed long non-coding RNA 1 (CCHE1) (Chen et al., 2017). Also, its high expression levels correlate with tumor size and poor survival prognosis in patients with this type of cancer. In a keratinocyte model that CCEPR is regulated by HPV16 E6 through a p53-independent mechanism (Sharma and Munger 2018). Interestingly, the viral E6 protein expression increased CCEPR, similar to HPV16-positive cervical cancer cells. In addition, the expression of both E6 and E7 further potentiates the expression of this lncRNA.

CCEPR localizes in the nucleus, where it probably interacts with proteins and nucleic acids rather than modulating PCNA levels in the cytoplasm (Sharma and Munger 2018). Notably, in oral squamous cell carcinoma (OSCC), CCEPR localizes to the nucleus and cytoplasm and acts as an oncogene to promote OSCC progression by increasing PAK2 expression through competitively binding miR-922 in OSCC cells (Figure 5) (Wang et al., 2020). Therefore, we consider it interesting to evaluate the presence of HPV in patients with OSCC to find out if this expression could also be related to the presence of the viral oncoproteins.

LncRNA-FAM83H-AS1 -lncRNA FAM83H-AS1 is overexpressed in cervical cancer cells and HPV16-positive head and neck cancer cell lines compared to HPV-16-negative cells (Barr et al., 2019). Notably, the presence of HPV16 correlates with elevated levels of FAM38H-AS1 from the early stages of carcinogenesis. FAM38H-AS1 is regulated by HPV through the presence of p300 in a p53-independent mechanism (Figure 5). Downregulation of this lncRNA impacts on proliferation, migration, and apoptosis (Barr et al., 2019).

LncRNA-FANCI-2 -It has been described that patients with aberrant expression of Fanconi anemia-linked genes have a high incidence of HPV-high risk-induced neoplasms. In a study by Haibin Liu et al. (2021) the authors showed that the expression of lnc-FANCI-2 and FANCI are positively co-expressed in HPV-18-infected cervical cancer cells (Liu et al., 2021). A cis-regulation of these genes has been proposed; however, the precise mechanism remains unknown. E6 and E7 oncoproteins inhibit miR-29-a expression, culminating in an increment in the expression of the transcription factor YY1. Interestingly, in CaSki cells, YY1 interacts with E7 to bind to the lnc-FANCI-2 promoter and induces transcriptional activation (Figure 3) (Liu et al., 2021).

LncRNA-EBIC -In cervical cancer cells, the expression of E6 and E7 stimulates the expression of EZH2-binding lncRNA (lncRNA-EBIC), also known as TMPOP2 (Thymopoietin pseudogene 2) (Sun et al., 2014; He et al., 2019). Mechanistically, EBIC interacts in the cell nucleus with EZH2, a central component of the PCR2 complex that catalyzes the H3K27me3 histone modification (Sun et al., 2014). Through this mechanism, lnc-EBIC exerts oncogenic properties as it participates in the silencing of E-cadherin to promote cell invasion and metastasis in cervical cancer.

The function of lnc-EBIC in the presence of HPV viral oncoproteins has been recently investigated. Hongpeng He et al. (2019) found that EBIC acts in the cytoplasm as an oncogene by inhibiting the function of some tumor suppressor miRNAs, i.e., it acts as an endogenous competitor of miR-375 and miR-139, whose function is to degrade E6 and E7 mRNAs (He et al., 2019). In this fashion, lnc-EBIC contributes to the tumoral phenotype of cervical cancer cells (Figure 5).

Regarding the transcriptional regulation of lnc-EBIC, p53-responsive elements were found in its promoter region. ChIP studies have shown a decreased enrichment of p53 in the promoter region of this lncRNA in some types of cancer (He et al., 2019). In addition, research by Jun Wang et al. has shown that exogenous expression of E7 from HPV16/18 significantly promotes lnc-EBIC expression, causing increased cell proliferation, migration, invasion, and inhibition of apoptosis through the TAL1/lnc-EBIC/KLHDC7B signaling axis (Wang et al., 2021).

LINC01101 y LINC00277 -Iancu et al. (2017) performed microarray assays based on E7 inhibition in HeLa (Iancu et al., 2017). The authors found many differentially expressed lncRNAs, including LINC01101 and LINC00277 (Figure 5), which were positively upregulated in the cells due to the lack of E7. In addition, they showed that high-risk HPV-positive samples have lower expression of LINC01101 and LINC00277 (Iancu et al., 2017). Moreover, low expression of LINC01101 correlated with FIGO stage and lymph node metastasis (Iancu et al., 2017).

The function and mechanism of action of LINC00277 (known as EWSAT1) appear to be tissue specific. For example, in Ewing’s sarcoma, EWSAT1 is positively regulated by EWS-FLI1 in primary human pediatric mesenchymal progenitor cells, and its high expression correlates with increased cell proliferation (Marques Howarth et al., 2014). Furthermore, Peng Song and Shu-Cheng Yin have shown that EWSAT1 is overexpressed in human nasopharyngeal carcinoma cells and correlates with poor survival (Song and Yin 2016). Mechanistically, it has been revealed that EWSAT1 indirectly regulates cyclin D1 expression, as it functions as a miRNA sponge for miR-326 and miR330-5p, which target cyclin D1 for degradation (Song and Yin 2016). Studies in osteosarcoma have shown that EWSAT1 is related to cellular phenomena such as proliferation, migration, invasion, and metastasis. As for its mechanism of action, EWSAT1 regulates the expression of the lncRNA MEG3 (Sun et al., 2016).

CRNDE -Studies by Harden et al. (2017), have suggested that the lncRNAs CRNDE (Colorectal Neoplasia Differentially Expressed), DANCR and TINCR, are transcribed due to HPV16 E6/E7 expression and could contribute to cancer development. For example, high expression of DANCR and low expression of TINCR could overlap with processes involved in epithelial differentiation (Harden et al., 2017; Sharma and Munger 2020c).

We believe that it would be essential to delve deeper into the regulation of the expression of these lncRNAs and their association with HPV, as they exert a very prominent role in cancer regulation. For example, lnc-CRNDE has multiple functions in the cell. For instance, it could function as ceRNA, RNA-decoy, scaffold and miRNA sponge in various types of cancer (Lu et al., 2020). Even lnc-CRNDE has been highlighted for its role in epigenetic regulation because it interacts with EZH2 to repress DUSP5 and CDKN1A. Specifically, in cervical cancer, which is a cancer type that develops due to HPV persistent infection, Ding et al. (2017) have shown that lnc-CRNDE is overexpressed in cervical cancer tissues and functions as an oncogene since its expression correlates with increased cell proliferation, advanced FIGO stage and poor patient prognosis (Ding et al., 2017).

As for its mechanism of action, in cervical cancer CRNDE binds to PUMA, a modulator of apoptosis, to inhibit its expression. As a result, there is an increase in the expression of Bcl-xL, an anti-apoptotic protein that plays a crucial role in cell death (Figure 5). Interestingly, Bcl-xL is overexpressed in patients with chemoresistance and shorter disease-free intervals (Zhang and Fan 2019).

Further studies in cervical cancer by Lu Ren et al. (2021) determined that CRNDE is a sponge for miR-4262, and interestingly, this miRNA targets ZEB1, a regulator of epithelial to mesenchymal transition (Figure 5). Thus, the CRNDE/miR-4262/ZEB1 signaling axis accelerates cervical cancer progression by increasing cell survival, EMT, migration, and invasion (Ren et al., 2021).

lncRNA-SNHG12 -The lncRNA-SNHG12 (*Homo sapiens* small nucleolar RNA host gene 12) has been studied in several types of cancer; however, studies by Lai et al. (2020), show that SNHG12 is highly expressed in CC cells infected with HPV. Functional assays showed that SNHG12 contributes to the malignant behavior of CC since SNHG12 participates in the activation of the ERK/Slug signaling pathway, which leads to the promotion of epithelial-mesenchymal transition, an increase in proliferation, invasion, and inhibition of apoptosis (Lai et al., 2020). HPV16-E6 and E7 proteins can drive SNHG12 expression through a c-Myc activation-dependent mechanism; however, the specific mechanism has not been fully described (Figure 5).

#### 5.2.2 Suppressing Long Non-Coding RNAs

LncRNA-DINO -Recent studies show that the Damage Induced lncRNA (DINO) is a 951 nucleotides long lncRNA located on chromosome six and divergently transcribed to the CDKN1A gene (Schmitt et al., 2016). Schmitt and coworkers have shown that DINO is transcribed under p53 regulation upon DNA damage stimuli and other stress factors using human cancer cell lines. DINO, once transcribed, forms a complex with p53 to increase its stability and enhance the signaling of this protein (Schmitt et al., 2016).

Interestingly, it has been demonstrated that DINO expression is increased in cells expressing the HPV-16 E7 protein (Sharma and Munger 2020b). It is also a critical mediator of p53 stabilization and activation. It has become evident that HPV16-E7 positive cells are more sensitive to cell death under various metabolic stress conditions; this association has been attributed mainly to p53 activation (Figure 5) (Sharma and Munger 2020b).

Additionally, DINO expression presents a regulation mediated by epigenetic derepression (H3K27 demethylation) through KDM6A. In addition, in cervical cancer cells, it has been shown that DINO activation occurs through a pathway that is independent of ATM/CHK2 (Sharma and Munger 2020a). Recent studies show that DINO is silenced in human cancers (Liu et al., 2019; Marney et al., 2021), and some evidence shows that a region of the DINO/CDKN1A locus is hypermethylated, leading to the silencing of DINO but not CDKN1A (Marney et al., 2021). Furthermore, silencing DINO in a model of primary HFKs E7+ HPV16+ using shRNAs decreases sensitivity to doxorubicin (Sharma and Munger 2020b).

Studies by Marney et al. (2021) have shown that loss of one or two DINO alleles alters the p53-mediated signaling and apoptosis pathway, resulting in an inefficient tumor suppressor phenotype. These insights are of great interest as new therapeutic strategies could aim to activate p53 through the reversal of epigenomic silencing of DINO.

LncRNA-PRINS -To understand HPV-linked HNSCC progression, Magda Kopczynska et al. (2020) performed analyses using data from The Cancer Genome Atlas (TCGA) (Kopczynska et al., 2020). In this analysis, the authors included 81 HPV (−) and 40 HPV (+) samples from patients with HNSCC. The results showed differentially expressed lncRNAs in HPV (+) such as PRINS, CDKN2B-AS1, TTTY14, TTTY15, MEG3, and H19 vs. HPV (−) patients (Kopczynska et al., 2020).

Interestingly, the Psoriasis susceptibility-related RNA Gene Induced by Stress lncRNA (lnc-PRINS) was overexpressed in HVP (+) patients and was associated with better overall survival and disease-free survival. Lnc-PRINS expression levels correlate with antiviral response genes, e.g., CRTSS, TLR8, IRF5, CCL5, CD40, NOD, CARD9, PYCARD, PSTPIP, and IFNAR1. In addition, lnc-PRINS modulates gene expression of transcripts involved in the inflammatory response, growth factors, proinflammatory genes, chemokines, and immunostimulators among others, so lnc-PRINS may stimulate the immune response of patients with HPV + HNSCC (Kopczynska et al., 2020). Some studies have indicated that tumors from HPV (+) patients have an increased number of immune cells, which may explain why HPV + HNSCC patients have a better response to therapy than HPV-negative patients (Figure 5).

## 6 Implications for Triage and Prognosis

DNA methylation status has been proposed in CC screening programs as a triage strategy for women with positive HPV results and cases with atypical squamous cells of undetermined significance (ASCUS). If not diagnosed and treated on time, the HPV persistent infection can generate precursor lesions that become cervical cancer. For this reason, once HPV+ cases have been identified in the screening programs, a second test (triage) should be carried out to differentiate between cancer precursor lesions that could potentially progress (persistent infection) from those that do not (transient infection). At this second triage, determining DNA methylation of a group of cellular or viral genes has enormous diagnostic utility for HPV (+) cases.

Initial studies postulated the methylation of CADM1 and MAL to detect cervical intraepithelial neoplasia (CIN) 2 and 3, being more sensitive as a triage strategy than cytology (Verhoef et al., 2015). Methylation of the transcription factor ZNF582 is associated with the presence of CIN3 in ASCUS patients (Liou et al., 2015), and ZNF582, together with PAX1, showed high sensitivity and specificity for HSIL detection (Tian et al., 2017). Several genes have been proposed whose methylation is a potential biomarker of precursor lesions; among them, we found POUF4 (Pun et al., 2015; Kocsis et al., 2017), ASCL1, LHX8, and ST6GALNAC5 (Verlaat et al., 2018).

Among the clinical studies that validate methylation profiles as biomarkers of CIN2, CIN3, and CC, is the S5 trial. S5 evaluates the methylation of EPB41L3 and the late region of HPV16, −18, −31, and −33 with a sensitivity of 74% and a specificity of 90% (Lorincz et al., 2016; Cook et al., 2019). Additionally, in cases with ASCUS, S5 can discriminate between low-grade (>CIN1 LSIL) and high-grade (CIN2+, HSIL) lesions (Hernandez-Lopez et al., 2019; Gu et al., 2020). Recently, a multicenter analysis demonstrated a sensitivity of 99.8% for detecting CIN3 and invasive carcinoma (Banila et al., 2022). The EPB41L3, L1, L2, and E2 (HPV16) panel has also been evaluated for the early diagnosis of oropharyngeal carcinoma. The use of his panel has a sensitivity of 70% and a specificity of 91%, and it only requires a gargle sample (Giuliano et al., 2020).

Subsequent studies confirmed the usefulness of EPB41L3, SOX1, and HS3ST2 as a triage method for HSIL detection (Clarke et al., 2017). Studies in the Latin American population have modified the cut-off point of this assay (3.1, 3.7) to improve sensitivity and specificity (Hernandez-Lopez et al., 2019; Ramirez et al., 2021).

Two panels of methylation markers to detect CIN2+ have been validated in the Dutch and Chinese populations (C13ORF18/EBP41L3/JAM3 y C13ORF18/ANKRD18CP/JAM3). These assays showed a sensitivity of 79% and 76% and a specificity of 57% and 65%, respectively (Li N et al., 2021). Other studies evaluated the ASCL1, LHX8, ST6GALNAC5, GHSR, ZIC1, and SST genes. Interestingly, the ASCL1/LHX8 genes showed greater specificity in identifying HPV-HR+ CIN3 with a similar sensitivity to HPV16/18 genotyping (Dick et al., 2020; Verhoef et al., 2022). This panel has been evaluated in urine samples to discriminate between LSIL and HSIL, managing to distinguish non-tumor tissue and low-grade lesions versus high-grade lesions, showing promising results, so it is suggested as a strategy to increase screening and triage (Van Keer et al., 2021).

Furthermore, long-term studies (14 years) showed that the cumulative incidence of CC in HPV+ samples is higher using FAM19A4 and miRNA-124 methylation profiles compared with a positive cytological result as a triage strategy (Luttmer et al., 2016; Clarke et al., 2017; Del Mistro et al., 2017; De Strooper et al., 2018; Dick et al., 2019). This assessment indicates that a negative result in FAM19A4/miR124-2 methylation could rule out the existence of CC (Vink et al., 2021).

Recent studies show that the FAM19A4/miR124-2 assay performed better than HPV16, 18, 31, 33, and 45 genotyping in HPV-positive women with borderline or moderate dyskaryosis, ASC, or LSIL, since the absolute risk of CIN3+ was higher (33%) than with other methods. Interestingly, combining the FAM19A4/miR124-2 assay with HPV16/18 increased detection by 40%. This strategy would allow more significant differentiation of patients with CIN3+ (Dick et al., 2022). Additionally, the methylation profile of FAM19A4/miR124-2 is constant in lesions caused by HPV 16 and 18 or by other high-risk genotypes (Leeman et al., 2018; Leeman et al., 2019). These genes were evaluated in European multicenter studies and have been assessed as a good triage option and an alternative to cytology (Bonde et al., 2021).

Other miRNAs such as miR-15b-5p and miR-375 have been evaluated together with FAM19A4, and this approach manages to distinguish CIN3 cases (Babion et al., 2018). In addition, the panel: C13orf18, EPB41L3 and JAM3, was validated for the CIN3 and CC detection (van Leeuwen et al., 2019). Assays for detecting malignant neoplasms associated with HPV-HR (+) based on epigenetic signatures are commercially available, for instance, GynTect® and QIAsure®. GynTect® is a molecular test for cervical carcinoma that analyzes the methylation profiles of 6 markers (ASTN1, ZNF671, DLX1, ITGA4, RXFP3, SOX17) (Schmitz et al., 2017; Schmitz et al., 2018). QIAsure® is a multiplex real-time methylation-specific PCR-based (qMSP) assay that detects hypermethylation of FAM19A4 and mir124-2 (Floore et al., 2019). It should be noted that the panel is consistent in CC, even with rare histotypes and negative HPV-HR carcinomas (Vink et al., 2019). A comparative study of both kits showed that GynTec has higher specificity than QIASure to detect CIN2+ (87% vs. 67%) and CIN3 (84% vs. 68%) (Dippmann et al., 2020).

Other commercially available panels for methylation evaluation are Precursor M Gold (CADM1, MAL, and miR124-2), CONFIDENCE Marker (POU4F3), and CERVI-M (PAX1) (Kremer et al., 2021). PAX1, in particular, has been evaluated in the Taiwanese population, finding comparable performance with cytology and better specificity than HPV16/18 genotyping as a triage strategy for the detection of CIN3+ in HPV-HR+ women (Chang et al., 2021). Interestingly, changes in viral DNA methylation have been associated with the transition from transient to persistent infection associated with cancer precursor lesions. Also, several studies have proposed the methylation of L1/L2 regions of carcinogenic HPV type 1 for the CIN3 and adenocarcinoma *in situ* detection (Clarke et al., 2018).

A meta-analysis identified the DNA methylation of CADM1, MAL, MIR-124-2, FAM19A4, POU4F3, EPB41L3, PAX1, SOX1, and L1/L2 from HPV16 as a triage strategy for HPV-AR+ cases (Kelly et al., 2019).

These studies indicate that in CC, the HPV test and triage based on DNA methylation, identify with higher specificity and sensitivity, healthy vs. precursor lesions. All those methylation biomarkers validated, could be extended to other HPV (+) neoplasms to early detection.

## 7 Epigenetic Therapy and Chemoprevention

The dynamic and reversible nature of epigenetic modifications makes them ideal therapeutic targets for treating HPV-associated cancers. Several studies have analyzed the effect of epigenetic therapies and chemopreventive agents in both cell lines and HPV+ neoplasms, including CC and HNSCC.

The potential of different natural compounds derived from plant sources such as phytochemicals has been evaluated as chemopreventive or adjuvants in anticancer therapies for several neoplasms. Exploring the potential therapeutic use of these compounds is particularly important since there are no specific treatment strategies for HPV + HNSCC.

Accumulating evidence suggests that dietary intake of vitamin B and folates have preventive effects against CC (Piyathilake et al., 2014; Mahmoud and Ali 2019). Another therapeutic resource for HPV neoplasms is the systemic administration of retinoids such as vitamin A (Oren-Shabtai et al., 2021), due to the retinoid deficiency is associated with the methylation of the retinoic acid receptor β 2 (RARβ2), a tumor suppressor. In a transgenic mouse model, it was shown that the expression of HPV E7 and the lack of dietary retinoids induce proliferation, hyperkeratinization, development of precursor lesions, and cancer *in situ* (Ocadiz-Delgado et al., 2021).

Analyses using molecular modeling have predicted that genistein, a naturally occurring compound found in soybean, interacts with several DNMT and HDAC family members. This compound reduces the expression and enzymatic activity of DNMTs and HDACs in Hela cells. Genistein is also known to activate the expression of tumor suppressor genes such as MGMT, RARb, p21, E-cadherin, DAPK1, FTHI, RUNX3, CDH1, PTEN, SOCS51, and other genes related to cell cycle regulation, migration, and inflammation such as CCNB1, TWIST1, MMP14, TERT, AKT1, PTPRR, FOS, and ILIA (Sundaram et al., 2018; Sundaram et al., 2019). These data suggest that genistein could have chemopreventive and chemotherapeutic properties since it exhibits anti-inflammatory, antiangiogenic, antiproliferative, and pro-apoptotic activities.

Curcumin (diferuloylmethane), a polyphenol found in the rhizome of curcuma plants, has been reported to inhibit the transcription of E6 and E7, thus restoring the expression of p53, pRB, and PTEN (Maher et al., 2011). In CC cell lines, it was shown that curcumin improves paclitaxel-induced apoptosis through the NF-κB-p53-caspase-3 (Dang et al., 2015). Curcumin also suppresses proliferation and exhibits anticancer effects in oral squamous cell carcinoma (OSCC). Interestingly, this compound can chemo-sensitize CSCs by abrogating stemness and inhibiting growth more effectively in HVP+ cells. These pieces of research propose that curcumin is a strong candidate for therapeutic approaches, especially in HPV+ tumors (Bano et al., 2018). Mechanistically, curcumin also acts by inhibiting HDAC 1, 2, 3, 4, 5, 6, 8, and 11, resulting in increased histone acetylation levels (Soflaei et al., 2018).

The FDA has approved several HDAC inhibitors as therapeutics with anticancer potential. Accumulating evidence has demonstrated that HDAC inhibitors are helpful for the treatment of hematological malignancies such as multiple myeloma and leukemia. Meanwhile, the efficiency of HDAC inhibitors in solid tumors is currently being assessed.

*In vitro* assays show that HDAC inhibitors such as sodium butyrate and trichostatin A can reverse the HPV-induced proliferation. These inhibitors neutralize the viral oncoproteins, increasing p21 and p27 expression, resulting in the Cyclin-dependent kinases suppression activity, arrest in the G1 phase of the cell cycle, and therefore, p53-independent and p73-dependent apoptosis is induced (Finzer et al., 2001; Finzer et al., 2004). Also, in CC cell lines showed that hydralazine and valproic acid exhibited an antiproliferative effect dependent on upregulation and stability of p53 by acetylation. However, they also increased the expression of E6/E7 (de la Cruz-Hernandez et al., 2007). Interestingly, when these drugs were administered to patients, the expression of viral oncoproteins was not increase (NCT00404326). Later, it was identified that combining both drugs increased radiation cytotoxicity, enhanced by cisplatin (Mani et al., 2014). Subsequent phase III clinical trials in patients with advanced CC showed a more prolonged median survival (10 months) when treated with chemotherapy + valproic acid + hydralazine than chemotherapy + placebo (6 months). However, toxicities such as thrombocytopenia were higher in the experimental group (Coronel et al., 2011). Other HDACs inhibitors such as vorinostat, belinostat, and panobinostat reduce E6 and E7 activity resulting in apoptosis and abrogating viral replication (Banerjee et al., 2018).

It has been postulated that the failure of HDAC inhibitors is due to the activation of the LIFRα oncogenic signaling pathways since EC359 (inhibitor of LIFRα) attenuates side effects provoked by HDAC inhibitors (Li M et al., 2021). Another aspect that should be further evaluated is the epigenetic classification of tumors and their relationship with the response to HDAC inhibitors.

Regarding the combination of HDAC inhibitors with antivirals to eradicate HPV infections, hitherto, there are no HPV-specific antiviral agents approved for clinical use, however, machine learning and therapeutic repositioning are emerging strategies to identify antiviral molecules. Lin et al. (2021) identified 57 antiviral drugs with potential interaction with E1, E2, E4, E5, E6, E7 L1, and E8^E2 (Lin et al., 2021). However, the effectiveness of these candidates and their impact on epigenetic alterations have not yet evaluated.

In addition, HDAC inhibitors have been used in combination with other drugs that block the activity of immune checkpoint proteins, for example, Chidamide is a benzamide that selectively suppresses the activity of class I and class IIb HDACs, increasing the acetylation of histones H3/H4, thus promoting transcription. Chidamide is currently in phase Ib/II clinical trials (NCT04651127). This inhibitor sensitizes advanced and metastatic, persistent, or recurrent cervical cancer tumors for further treatment with PD-1 inhibitors to boost the immune response against cancer cells.

In transformed follicular lymphoma, chidamide impacts on the PI3K-AKT pathway (Zhong et al., 2021) and in lymphoma it causes G0/G1 arrest, declining the cyclin D and c-myc expression, and increasing p53 and p21. Additionally, chidamide induces caspase-dependent apoptosis (Yuan et al., 2019).

PEVO trial evaluates the response to the combination of pembrolizumab and vorinostat, HDAC inhibitor, in several tumors recurrent and metastatic squamous cell carcinomas, HPV+ vs. HPV−. This study might provide clues to determine how HPV presence influences tumors’ therapeutic response and could also identify predictive and pharmacodynamic biomarkers (de Guillebon et al., 2021).

Other therapies include the epigenetic factor DNMT. 5-azacytidine and decitabine are drugs approved by the FDA to inhibit DNMTs for myelodysplastic syndrome and acute myeloid leukemia treatment. In HPV + solid tumors, has been evaluated the potential of these drugs as monotherapy or in combination with chemotherapy or immunotherapy.

In murine models and clinical trials in HNSCC HPV+ patients (NCT02178072), the 5-azacitidine treatment decreases proliferation, expression of viral genes, the activity of metalloproteases and induces p53-dependent apoptosis (Biktasova et al., 2017). In another clinical trial, intravenous administration of decitabine was evaluated for patients with HPV+ HNSCC anogenital carcinomas at high risk of recurrence, after radiotherapy (NCT04252248). However, there are still no conclusive results from this trial. Interestingly, it has been proposed to evaluate the effect of orally administered decitabine, in combination with durvalumab for patients with oral cavity, oropharynx, hypopharynx, or larynx recurrent or metastatic carcinomas. In this clinical trial, the response in HPV+ vs. HPV− patients will be compared, evaluating the APOBEC expression, the interferon pathway activation, proliferation, and apoptosis (NCT03019003).

An additional epigenetic factor of great relevance is EZH2. Since EZH2 is more expressed in HPV+ OPSCC tumors than in HPV− cases (Idris et al., 2016), there are therapies employed to inhibit EZH2, such as siRNAs, 3-deazaneplanocin A, GSK-343, and EPZ005687. Immunotherapy has undoubtedly improved the life expectancy of patients with malign neoplasms such as melanoma. Nevertheless, there are still several types of tumors where the impact of immunotherapy remains to be proven. The combination of immunotherapy and epitherapy has recently been proposed in different neoplasms.

The epigenetic effects of chemopreventives and therapeutic agents in HPV-associated neoplasms are still a subject of study from a functional and clinical perspective.

## 8 Final Remarks

Unlike genetic alterations, epigenetic alterations are potentially reversible. Epigenetic, systemic or targeted therapy is an area with great potential for the treatment of several neoplasms. These therapies must be directed according to the biological characteristics of tumor, in order to provide the greatest benefit and reduce adverse effects. Epigenetic alterations are a tool for monitoring therapeutic responses. The control of neoplasms associated with HPV is a feasible challenge to achieve since there are prophylactic vaccines, screening, and triage methods, which are focused on cervical cancer, however, in other neoplasms these strategies can also have an impact favorable. The discovery of epigenetic alterations induced by HPV, and new viral non-coding RNAs and methylation signatures, opens the possibilities for their application as triage biomarkers, and for designing drugs to modify the epigenetic landscape for improved patient survival and quality of life based on comprehensive treatments.
